# A UMAT–UDSM interface for integrating Plaxis user soil models into Abaqus: application to the hardening soil model

**DOI:** 10.1016/j.mex.2026.104050

**Published:** 2026-07-15

**Authors:** Luis Mugele, Daniela Alejandra Villarreal Illada, Heiko Neher, Christoph Niklasch, Hans Henning Stutz

**Affiliations:** a Institute of Soil Mechanics and Rock Mechanics (IBF), Karlsruhe Institute of Technology (KIT), Engler-Bunte-Ring 14, Karlsruhe, 76131, Germany; b Ed. Züblin AG, Zentrale Technik, Albstadtweg 3, Stuttgart, 70567, Germany

**Keywords:** Umat.for, Udsm.for, Finite element method, Constitutive modeling, Elastoplasticity, Geotechnical engineering, Abaqus, Plaxis, Hardening soil model

## Abstract

Finite element (FE) programs such as Plaxis and Abaqus allow so-called user-defined constitutive models. However, each FE code requires the user-defined constitutive equations to be implemented in a different internal solver-specific format. This limits the reusability of the user-defined implementations within different FE codes, even when the theoretical formulation of the constitutive model is identical.

This paper presents an interface that couples a constitutive formulation originally implemented as a Plaxis user-defined soil model (UDSM) with an implementation in Abaqus through a user material subroutine (UMAT). Thus, a given UDSM can easily be used as a UMAT in Abaqus. The interface preserves the original constitutive implementation and therefore the parameter and state variable structure, enabling direct execution of the Plaxis-style UDSM within an Abaqus UMAT. The proposed method is demonstrated and validated using a UDSM that describes the Hardening Soil model (HSM) through element tests and a boundary-value problem solved in Abaqus and Plaxis, confirming its accuracy and numerical stability.

• UMAT interface for Abaqus that executes an external Plaxis-specific UDSM.
• Generic interface that enables the plug-in of other UDSM subroutines in Abaqus with minimal modification.
• Validation using the Hardening Soil model (HSM) via element tests and boundary value problem.

## Graphical abstract

**Figure:**
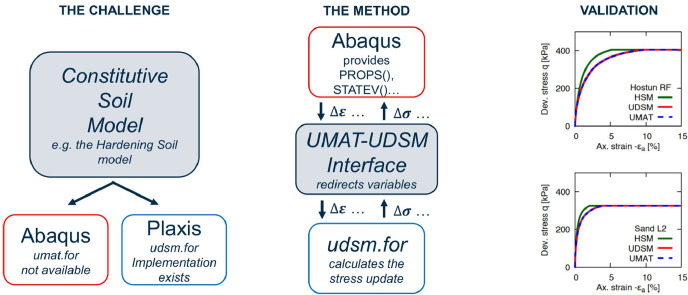

## Specifications Table

**Table:** 

**Subject area**	Engineering
**More specific subject area**	Soil mechanics and geotechnics, numerical analyses, constitutive modelling
**Name of your method**	Plaxis udsm.for to Abaqus umat.for interface
**Name and reference of original method**	None
**Resource availability**	https://doi.org/10.5281/zenodo.21453420

## Background

Finite element (FE) simulations represent the state of the art in the analysis of continuum-mechanical stress-strain behavior of structural components and in the geotechnical engineering of soil-structures and their soil-structure interaction. However, soil behaves unlike most engineering materials. Its mechanical behavior is highly nonlinear and depends, among other factors, mainly on the current stress state, the density, and the most recent deformation history [1,2]. Therefore, for high-quality stress and deformation predictions of geomaterials, for example, deformations of an excavation pit, advanced constitutive models should be used. Unfortunately, these advanced constitutive models for soil (for example, elastoplastic, hypoplastic, or bounding surface formulations) often exceed the in-house available constitutive models in commercial FE codes. Therefore, they usually provide interfaces that enable user-defined constitutive models to be integrated into the FE analyses.

Widely used FE codes in geotechnical engineering are Plaxis [3] and Abaqus [4], on which the proposed method within this work focuses. To integrate user-defined constitutive soil models, Plaxis uses the so-called user-defined soil model (UDSM) code through a compiled dynamic-link library, and Abaqus relies on the user material subroutine (UMAT), both written in Fortran. Both the UDSM and the UMAT enable users to define complex, non-linear material behavior on the integration point level, but their technical implementation differs substantially.

However, the UMAT and the UDSM operate in a conceptually similar manner. From the FE code they mainly receive a defined set of material parameters, the current state of the soil including the actual (effective)^1^ stress tensor σ and additional state variables S (for example void ratio, preconsolidation pressure, intergranular strain, fabric tensor, …), an incremental strain tensor Δε, and the corresponding time increment Δt from which they calculate the incremental stress response Δσ and the corresponding increment of the additional state variables Δα by integrating the constitutive equations within a defined numerical scheme. Additionally, they provide a local tangent stiffness matrix, the so-called Jacobian D, which is required by the respective FE code for equilibrium iteration within each increment. Other values exchanged between the UMAT or UDSM and the corresponding FE code are primarily used for numerical purposes and are of secondary relevance for the constitutive model. This structural similarity between the UMAT and the UDSM enables the possibility of creating a bidirectional interface between the two environments to overcome the aforementioned technical differences. Using the hereinafter presented method of a UMAT-UDSM interface, a UDSM originally written for Plaxis can be easily applied in Abaqus as UMAT.

Previous works, such as the UDSM-UMAT interface [5] published on Soilmodels, have already demonstrated the concept in reverse [5] provides an interface for running a UMAT via a UDSM in Plaxis. This example illustrates that the idea of establishing an interface between UMAT and UDSM is not only feasible but has already been successfully implemented in the opposite direction, thus providing a strong conceptual foundation for the present work. With the VUMAT-UMAT interface [6] published also on Soilmodels, a given UMAT can also be used in an explicit analysis in Abaqus.

## Method details

**Fig. 1:**
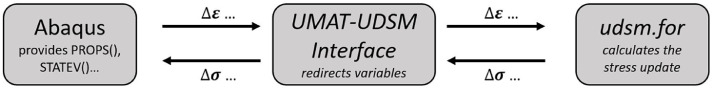
General and simplified procedure of the proposed UMAT-UDSM interface.

The present work introduces a UMAT-UDSM interface that enables an Abaqus UMAT to directly call and execute a Plaxis UDSM without changing the original implementation. The method uses a data exchange between both frameworks mainly by (i) mapping Abaqus strain increments and stress states to the input structure expected by the Plaxis UDSM; (ii) transferring the latter and the state variables between the UMAT´s array and the UDSM´s internal memory and (iii) converting the UDSM outputs (updated stress state, updated state variables and stiffness matrix) back into Abaqus quantities. The general and simplified procedure of the proposed UMAT-UDSM interface is shown in Fig. 1. Simply speaking, Abaqus provides for each UMAT call the material properties, the strain increment Δε, the time increment Δt, the current stress state σ, and the state variables. These arrays are reformatted and sent to the external UDSM subroutine, which performs the integration of the constitutive model and returns the updated stress tensor, the updated state variables, and the tangent stiffness matrix. The UMAT then transfers these results back to the Abaqus solver.

Note that the UDSM is always called using the effective stress. If an analysis with locally undrained conditions is performed, the total stress is decomposed into effective stress and pore water pressure, including the evolution equation of the latter, in the UMAT. Therefore, it is always necessary to specify the bulk modulus of the pore fluid divided by the porosity $K_{w}/n$ as props(2)^2^, and the evolving pore water pressure is stored in statev(1). For locally drained or dry conditions, $K_{w}/n=0$ must apply. Hydro-mechanically coupled problems under non-locally undrained conditions can likewise be addressed using the proposed UMAT–UDSM interface. In this case, the hydro-mechanical coupling is performed internally by Abaqus, whereas the UMAT–UDSM interface only supplies the constitutive mechanical response associated with the selected material model. The numerical parameter $p_{t}$, given via props(1) and referred to as numerical cohesion, shifts the stress tensor isotropically and can be used to overcome numerical issues related to very small effective stress conditions. Both UMAT and UDSM use the mechanical sign convention, so tensile stresses and strains are positive.

To facilitate understanding of the UMAT-UDSM interface workflow, the coupling procedure is divided into four main parts. These cover (1) the adaptation of Abaqus inputs, (2) the initialization of USER_MOD dummy arguments, (3) the execution of the constitutive model via the call of the UDSM, and (4) the conversion of outputs back into Abaqus format. These parts are described subsequently in detail using the corresponding part of the code at the UMAT level.

1. **Input adaptation:** In the first part, the Abaqus strain increment DSTRAN, total strain STRAN, and current stress STRESS are reorganized and mapped into the slightly different input structure expected by the UDSM [3,4]. The above-mentioned numerical cohesion $p_{t}$ and pore water pressure $p_{f}$ (if $K_{w}/n\;>0$, i.e., locally undrained calculation) are considered in the isotropic part of the stress. In addition, the material parameters props() and the state variables statev() of Abaqus are transferred in the respective arrays parms()^3^ and stvar() for the UDSM call, excluding the non-material model-related quantities $p_{t}$ and $K_{w}$/n, as well as $p_{f}$.

C ! Axis translation due to numerical cohesion (p_t > 0)

C ! Calculate effective stresses

PORE = STATEV(1)

PT = PROPS(1)

BULK_W = PROPS(2)

SIG0(1) = STRESS(1) - PT + PORE

SIG0(2) = STRESS(2) - PT + PORE

SIG0(3) = STRESS(3) - PT + PORE

SIG0(4) = STRESS(4)

IF (NTENS .EQ. 6) THEN !Check ntens

SIG0(5) = STRESS(6)

SIG0(6) = STRESS(5)

ENDIF

C ! Constitutive Parameters

DO I = 1, NPROPS-2

PARMS(I) = PROPS(I+2)

ENDDO

C ! Strain increment

DO I = 1, 4

DEPS(I) = DSTRAN(I)

ENDDO

IF (NTENS .EQ. 6) THEN

DEPS(5) = DSTRAN(6)

DEPS(6) = DSTRAN(5)

ENDIF

C ! State variables

DO I = 1, NSTATV-1

STVAR0(I) = STATEV(I+1)

ENDDO

2. **Initialization of dummy arguments for USER_MOD:** The subroutine USER_MOD within the UDSM framework serves as the main entry point of the UDSM and manages all input-output operations within the UDSM. Before execution, a set of dummy arguments must be initialized to ensure compatibility. This includes parameters defining the model number, step counters, element and integration point identifiers, as well as numerical flags controlling stress dependency and time effects^4^.

Certain parameters within the UDSM framework, such as the bulk modulus of water (BULKW) and the pore pressure (SWP), are set to zero because local undrained conditions are handled directly within the UMAT rather than inside the UDSM, and the UDSM is only used in terms of effective stresses. The array IPRJDIR specifies the file storage path used by the UDSM during processing. In this implementation, the path corresponds to D:\, defined using ASCII codes and a path length variable (IPRJLEN), which can be easily modified by the user.

C ! Variable initialization for User_Mod

MODEL = 3 ! This should be modified: Apply the model number that is being used

ISUNDR = 0

ISTEP = 1

ITER = 1

IEL = NOEL

INTP = NPT

X = COORDS(1)

Y = COORDS(2)

Z = COORDS(3)

TIME0 = TIME(1)

SWP = 0.0D0

BULKW = 0.0D0

NSTAT = 2 ! This should be modified: Apply the information used in UDSM for IDTask = 5

NONSYM = 0 ! This should be modified: Apply the information used in UDSM for IDTask = 5

ISTRSDEP = 1 ! This should be modified: Apply the information used in UDSM for IDTask = 5

ITIMEDEP = 0! This should be modified: Apply the information used in UDSM for IDTask = 5

ITANG = 0! This should be modified: Apply the information used in UDSM for IDTask = 5

IABORT = 0

C ! This should be modified if another path is wished for. Current path: D:\

IPRJLEN = 3 ! Length of the array IPRJDIR (number of characters in the path)

IPRJDIR(1) = 68! ASCII code of the character 'D'

IPRJDIR(2) = 58! ASCII code of the colon ':'

IPRJDIR(3) = 92 ! ASCII code of the backslash '\'

3. **Call for USER_MOD:** Once all necessary variables and parameters are initialized, the USER_MOD subroutine of the UDSM is called. This routine performs the constitutive calculations based on the specified task identifier (IDTASK). Two separate calls are made:

For IDTASK = 3, USER_MOD computes the components of the tangent stiffness matrix (Jacobian).

For IDTASK = 2, it updates the stress tensor and the corresponding internal state variables.

C———————————————————————

C———— compute and store stiffness matrix ——

C———————————————————————

C Call User_Mod for stiffness matrix

IDTASK = 3

CALL USER_MOD(IDTASK, MODEL, ISUNDR, ISTEP, ITER, IEL, INTP,

+ X, Y, Z, TIME0, DTIME,

+ PARMS, SIG0, SWP, STVAR0,

+ DEPS, D, BULKW,

+ SIG_END, SWP, STVAR, IPL,

+ NSTAT, NONSYM, ISTRSDEP, ITIMEDEP, ITANG,

+ IPRJDIR, IPRJLEN, IABORT)

C———————————————————————-

C————— stress and state variable update ———–

C———————————————————————-

IF (.NOT. (KINC .EQ. 0 .AND. KSTEP .EQ. 1)) THEN

C Call User_Mod for stress update

IDTASK = 2

CALL USER_MOD(IDTASK, MODEL, ISUNDR, ISTEP, ITER, IEL, INTP,

+ X, Y, Z, TIME0, DTIME,

+ PARMS, SIG0, SWP, STVAR0,

+ DEPS, D, BULKW,

+ SIG_END, SWP, STVAR, IPL,

+ NSTAT, NONSYM, ISTRSDEP, ITIMEDEP, ITANG,

+ IPRJDIR, IPRJLEN, IABORT)

4. **Output translation and storage:** In the final stage, the updated stress tensor, tangent stiffness matrix, and internal variables returned by USER_MOD subroutine are translated back into the Abaqus format. These quantities are stored in the arrays STRESS, DDSDDE, and STATEV, ensuring their compatibility with the Abaqus solver.

To maintain consistency with the local undrained formulation, the pore pressure is updated based on the volumetric strain increment (DEPSV) and $K_{w}/n$. Further, in the case of locally undrained conditions, the resulting updated total stress tensor is determined using the updated effective stress and the updated pore pressure.

C ! ... update pore pressure and compute total stress

DEPSV = DEPS(1) + DEPS(2) + DEPS(3)

PORE = PORE - (BULK_W * DEPSV)

C ! ... update stresses and state variables

DO I = 1, NSTATV-1

STATEV(I+1) = STVAR(I)

ENDDO

DO I = 1, 3

STRESS(I) = SIG_END(I) + PT - PORE

ENDDO

STRESS(4) = SIG_END(4)

IF (NTENS .EQ. 6) THEN

STRESS(5) = SIG_END(6)

STRESS(6) = SIG_END(5)

ENDIF

STATEV(1) = PORE

C ! ... update jacobian

DO J=1,NTENS

DO I=1,NTENS

DstvarDSDDE(I,J) = D(I,J)

ENDDO

ENDDO

DO J=1,3

DO I=1,3

DDSDDE(I,J) = DDSDDE(I,J) + BULK_W

ENDDO

ENDDO

The state variables are preferably initialised in Abaqus using the subroutine sdvini.for, whereas in Plaxis the state variables are initialized using IDTASK = 1. Typically, UDSMs therefore contain ‘parameters’ that are not constitutive and are just used to transfer initial conditions to IDTASK = 1. Such parameters are simply dragged through in the UMAT-UDSM interface, but their values are not considered in the calculation. The values can therefore be set to 0.

## Application for use in other external UDSM

The UMAT-UDSM interface can be extended to any Plaxis-style User Defined Soil Model (UDSM) by adapting a few key elements related to file format, property alignment, and state variable configuration. All parts of the interface that have to be adjusted in the UDSM changes are highlighted with the comment ”! This should be modified”. Some important details of the UMAT-UDSM interface are:

1. Ensure fixed-format Fortran compatibility: The UDSM source code must be written in Fortran fixed format to ensure proper compilation and compatibility. If the original UDSM was developed in free format, it can be easily adapted by adding the compiler directive: “!DEC$ FREEFORM” and saving the file using a fixed format extension (e.g., .for).
2. Adapt the number and alignment of material properties: Within the interface, the first two entries of the PROPS array are reserved for $p_{t}$ and $K_{w}/n$ . Starting from the third position, all remaining properties are read in the order defined within the UDSM. Therefore, the total number of material properties to be declared in the Abaqus input file must correspond to N_props_^UMAT^ = N_parms_^UDSM^ + 2.
3. Adapt the number of state variables: Similarly, the first entry of the STATEV array is reserved for pore pressure $p_{f}$, ensuring consistency with a local undrained formulation. The remaining state variables correspond directly to those used in the UDSM. Consequently, the total number of state variables required by the UMAT is N_STATEV_^UMAT^ = N_STVAR_^UDSM^ + 1.
4. Apply the appropriate model number: In many UDSM frameworks, the USER_MOD subroutine can include several constitutive models identified by a model number parameter (IMOD). This parameter determines which constitutive law is activated during execution. When coupling a specific UDSM to the UMAT, the model number must be adjusted to match the desired implementation within the USER_MOD framework.
5. The names of the individual included Fortran files of the UDSM framework must be modified in the interface.

These minimal adaptations allow the interface to execute any constitutive model originally developed for Plaxis without altering the internal logic of its stress-strain integration scheme. If convergence issues occur during simulations, a small numerical cohesion value (e.g, $p_{t}=1$kPa) can be introduced to improve numerical stability.

The UMAT-UDSM interface can be found in the download package, along with documentation and an example Abaqus input file. Integrating the interface into a finite element analysis is as straightforward as using an user material subroutine UMAT in Abaqus.

## Method validation

To validate the proposed method, a UDSM implemented in a Fortran file is required. In principle, any constitutive model could be employed for this purpose. However, one material model is of particular practical relevance: the widely used Hardening Soil Model (HSM).

The HSM is extensively applied in geotechnical engineering and is available as a native material model in Plaxis. However, no corresponding freely available implementation currently exists for Abaqus. Therefore, the HSM is adopted in the following as an illustrative and representative case study within the broader UMAT–UDSM interface plug-in architecture. This demonstrates both the general applicability and the practical value of the proposed interface. For this purpose, a UDSM by Castellón [7], which approximates the well-known Hardening Soil model (HSM), is used hereafter. The basic concepts of the original HSM are briefly reviewed first.

### On the Hardening Soil Model (HSM)

The HSM is an advanced elasto-plastic constitutive model developed mainly by Schanz [8] and Schanz et al. [9] to describe the nonlinear stress-strain behavior of both cohesive and granular soils. The HSM uses a stress-dependent stiffness, with different formulations for virgin loading and unloading/reloading. Plastic strains are obtained by means of a multi-surface yield criterion. Isotropic hardening is assumed, governed by both plastic shear and volumetric strains. Frictional hardening follows a non-associated flow rule, whereas cap hardening is described using an associated flow rule. The model was originally implemented in Plaxis, which is widely used for deformation and stability analyses in soil mechanics [3].

In the HSM, irreversible compressive volumetric strains are associated with the expansion of the yield surface (volumetric hardening) governed by the evolution of the first additional state variable of the so-called preconsolidation pressure $p_{p}$, while shear hardening is described using the irreversible deviatoric strains described by the second additional state variable of the so-called plastic shear strain $\gamma_{p}$. Overall, the HSM uses, in addition to the effective stress tensor σ, two state variables ($p_{p}$ and $\gamma_{p}$). In Plaxis, the state variables are usually initialized using the soil weight γ, the earth pressure coefficient $K_{0}^{\text{nc}}$, and the overconsolidation ratio OCR.

The stiffness is defined by three reference moduli: the secant modulus in standard drained triaxial loading $E_{50}^{\text{ref}}$, the unloading-reloading (elastic) modulus $E_{\text{ur}}^{\text{ref}},$ and the oedometer modulus $E_{\text{oed}}^{\text{ref}}$. Each of these moduli varies with the effective confining pressure following a power law with an exponent m corresponding to a reference pressure $p_{\text{ref}}$ and a Poisson ratio $\nu_{\text{ur}}$ for the elastic un- and reloading stiffness. The strength is controlled via the friction angle φ and the cohesion c. The earth pressure coefficient $K_{0}^{\text{nc}}\;$(stress ratio of the horizontal effective stress to vertical effective stress in a normally consolidated state) is used to estimate the oedometric stiffness via the internal parameters discussed later, as well as to define the initial stress state via the Plaxis initialization procedure. Further parameters are the dilatancy angle ψ and the failure ratio $R_{f}$. In addition, the tension cut-off $\sigma_{T}$ is sometimes used. Details can be found in [[8], [9], [10], [11]].

Interestingly, the HSM has three additional internal parameters: the initial secant stiffness $E_{i}^{\text{ref}}$_,_ the cap steepness α, and the cap parameter (stiffness ratio) $K_{s}/K_{c}$ [10], which can generally be determined iteratively through the simulation of a drained triaxial test and an oedometer test, and are correlated with other reference parameters such as $E_{50}^{\text{ref}}$, $E_{\text{oed}}^{\text{ref}}$ and the earth pressure coefficient $K_{0}^{\text{nc}}$. The internal parameters are not defined by the user in Plaxis but are calculated internally by Plaxis. To overcome this plaxis-based and black-box-like estimation of the internal parameter, Castellón [7] provides an iterative procedure for the estimation of these internal variables.

A practical procedure for the determination of $E_{i}^{\text{ref}}$, α, and $K_{s}/K_{c}$ is also to use the Plaxis automated calibration tool itself. In the first step, triaxial and oedometer tests are calculated with the original HSM in the Plaxis element driver, and the corresponding curves are exported. In the second step, this data is then imported into the automatic calibration tool within Plaxis as test data, and the internal HSM parameters of the UDSM (HSM approximation), which are normal parameters within the UDSM, are determined using the automatic calibration tool from Plaxis.

Note that the quantities $K_{0}^{\text{nc}}$ and $\sigma_{T}$, required as input in the HSM of Plaxis, do not actually represent a parameter in the sense of a material model. $K_{0}^{\text{nc}}$ is used to estimate the aforementioned internal parameters, and $\sigma_{T}$ controls the tension cut-off criterion [10].

Since the internal implementation of the HSM in Plaxis is proprietary and not publicly available, Castellón [7] developed an independent implementation of the HSM in the form of a UDSM written in Fortran for use in Plaxis. This allows an external and transparent implementation of the constitutive model within the Plaxis software and provides a complete numerical scheme for integrating the equations. Note that Castellón´s UDSM was created to support the development of the so-called Elasto-Plastic Hysteretic Small Strain Model (EPHYSS) [7]. Castellón [7] already validated his UDSM for the HSM using a series of element tests within the Plaxis code, showing a good agreement with the original HSM.

However, the widely used HSM is still not available in Abaqus. Therefore, in the present study, Castellón´s UDSM is further used as the base implementation for the proposed UMAT-UDSM interface. This allows the proposed method to be validated and, at the same time, makes the HSM accessible in Abaqus. In addition to the interface itself, this work therefore offers a further advantage, as HSM implementations for Abaqus are currently only sparsely available.

Note that minor modifications of the numerical implementation of the UDSM by Castellón not affecting the constitutive formulation (for example, adapting it to the fixed-format Fortran requirements of Abaqus) were required.

The proposed UMAT-UDSM interface is validated using the UDSM implementation of the HSM provided by Castellón [7] based on (i) element tests and (ii) a boundary value problem (BVP). For comparison, the original HSM from Plaxis, the UDSM by Castellón [7] in Plaxis, and the UMAT with the proposed interface in Abaqus are compared and referred to as HSM, UDSM, and UMAT subsequently. The element tests of the UMAT are conducted for simplicity using the freely available IncrementalDriver software [12].

## Constitutive parameters

The parameter sets used for the validation are taken from Benz [10], specifically for Hostun RF sand and three sands (L1, L2, and L3) from an excavation in Berlin. The parameter sets published by Benz [10] are summarized in Table 1. Note that the last two entries should be understood as initial conditions rather than constitutive parameters. Each parameter set includes the corresponding internal parameters, allowing the UDSM and the UMAT to be used without further calibration.

**Table 1:** Constitutive parameters from Benz [10].

Parameter	Unit	Hostun RF	Sand L1	Sand L2	Sand L3
$E_{50}^{\text{ref}}$	kPa	30000	45000	75000	105000
$E_{\text{oed}}^{\text{ref}}$	kPa	30000	45000	75000	105000
$E_{\text{ur}}^{\text{ref}}$	kPa	90000	180000	300000	315000
m	-	0.55	0.55	0.55	0.55
c	kPa	0.0	1.0	1.0	1.0
φ	°	42.0	35.0	38.0	38.0
ψ	°	16.0	5.0	6.0	6.0
$\nu_{\text{ur}}$	-	0.25	0.2	0.2	0.2
$p^{\text{ref}}$	kPa	100.0	100.0	100.0	100.0
$R_{f}=\;q_{f}/q_{a}$	-	0.9	0.9	0.9	0.9
$\sigma_{T}$	kPa	0.0	0.0	0.0	0.0
$E_{i}^{\text{ref}}$	kPa	65488	96662	154447	208642
α	-	1.47	1.48	1.87	1.88
$K_{s}/K_{c}$	-	1.84	2.15	2.07	1.59
$K_{0}^{\text{nc}}$	-	0.4	0.43	0.38	0.38
OCR	-	1	1	1	1

### Drained triaxial test

**Fig. 2:**
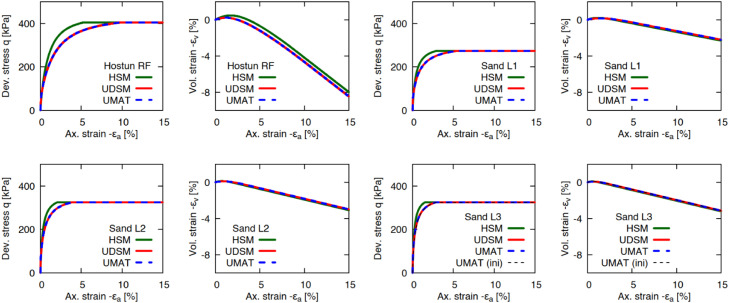
Simulated drained triaxial compression tests: Deviatoric stress q and volumetric strain $\varepsilon_{v}$ as a function of the axial strain $\varepsilon_{a}$ for Hostun RF sand and Berlin sands (L1, L2 and L3) implemented using HSM, UDSM, and UMAT.

Drained triaxial compression tests on normally consolidated sand are simulated for every parameter set with an initial isotropic stress state of $p_{0}=100\;\text{kPa}$. The resulting deviatoric stress $q=\sigma_{r}-\sigma_{a}$ and volumetric strain $\varepsilon_{v}=\varepsilon_{a}+2\varepsilon_{r}$ are plotted against the axial strain$\;\varepsilon_{a}$ in Fig. 2. Note that compressive stresses and strains are negative (mechanical sign convention).

To ensure consistent behavior between the HSM, the UDSM, and the UMAT, the state variables must be initialized equivalently. The initialization of the effective stress tensor is trivial and is set isotropically to $p_{0}=100\;\text{kPa}$. However, the initialization of $p_{p}$ and $\gamma_{p}$ must be discussed in detail. In the HSM, the initialization of these state variables cannot be controlled directly and is done internally in Plaxis using the stated values of $K_{0}^{\text{nc}}$ and OCR in Table 1. Note that the initial values of $\gamma_{p}$ and $p_{p}$ are not displayed and not accessible in the Plaxis SoilTest (Plaxis internal element driver) output.

In the UDSM used in Plaxis, this initialization is performed automatically through the internal routine defined by IDTask = 1, which computes the required variables from the described equations in the implementation from [7] using $K_{0}^{\text{nc}}$and OCR and the effective stress.

In contrast, the UMAT requires a manual initialization of the state variables. Therefore, the equations for the initialization within the UDSM are used to calculate the initial values for the UMAT calculation. Table 2 lists these initial values, ensuring that the UDSM and the UMAT calculations start with identical initial conditions. Note that in the examples shown, an intuitive initialization with $\gamma_{p}=0$ and $p_{p}=100\;\text{kPa}$ causes only minor deviations in the results of the simulations from the UMAT and UDSM. An additional calculation with this initialization for the sand L3 is shown in the bottom right of Fig. 2 and stated as UMAT (ini).

**Table 2:** Initial state variables used in the triaxial test simulations.

State variable	Unit	Hostun RF	Sand L1	Sand L2	Sand L3
$\gamma_{p}$	-	7.814 × 10^-4^	8.191 × 10^-4^	5.562 × 10^-4^	3.241 × 10^-4^
$p_{p}$	kPa	-108.009	-107.160	-105.353	-105.297

The results, shown in Fig. 2, exhibit an almost perfect agreement between the UMAT and the UDSM implementation, confirming that the UMAT is capable of reproducing the constitutive behavior described in the UDSM code. The results demonstrate the effectiveness of the UMAT-UDSM interface. Furthermore, compared to the HSM simulations, only minor deviations can be observed, which suggests that Castellón's UDSM closely replicates the original HSM. The differences are most pronounced for the Hostun RF material, although the qualitative behavior is similar. In practical application, these deviations are generally negligible. The discrepancies can be attributed to different initial conditions, different internal parameters, and small differences between the equations of the original HSM and those of Castellón’s UDSM. It should be noted that all simulations were performed using the same parameters from Table 1. A slight adjustment of the parameters would allow for an even better correlation between the curves.

### Undrained triaxial test

**Fig. 3:**
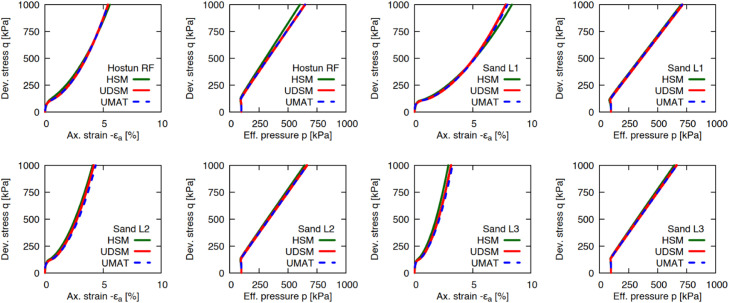
Simulated undrained triaxial compression tests: Deviatoric stress q as a function of the axial strain $\varepsilon_{a}$ and the mean effective pressure p for Hostun RF sand and Berlin sands (L1, L2, and L3) implemented using HSM, UDSM, and UMAT.

Undrained triaxial tests are simulated using the same initial effective stress state and initial values of the additional state variables used for the simulations of the drained triaxial compression tests. The resulting deviatoric stress q is shown in Fig. 3 as a function of the axial strain $\varepsilon_{a}$ and the mean effective pressure $p=\left(\sigma_{a}+2\sigma_{r}\right)/\left(-3\right)$. Note that an undrained triaxial test is simulated in the Plaxis element driver by prescribing the total stress path and considering the bulk modulus of the pore fluid, which leads to small volumetric deformations, whereas in the simulations with the UMAT using IncrementalDriver [12], a volume-preserving strain path is directly described. This conceptual difference has no practical implications for the shown simulations within this paper.

The results, shown in Fig. 3, demonstrate an excellent agreement between both the UDSM and UMAT, as expected from their identical constitutive formulations. Again, the deviations from the original HSM simulations are generally small.

### Oedometric compression test

**Fig. 4:**
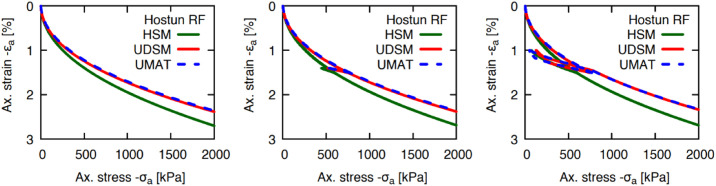
Simulated oedometric compression tests: Axial strain $\varepsilon_{a}$ as a function of the axial stress $\sigma_{a}$ for Hostun RF sand implemented using HSM, UDSM, and UMAT, considering a pure monotonic loading (left), a small (center), and a large (right) un- and reloading.

Oedometric compression tests on normally consolidated sand are simulated for Hostun RF sand with an initial isotropic stress state of $p_{0}=1\;\text{kPa}$. For simplicity, $p_{p}=1\;\text{kPa}$ and $\gamma_{p}=0$ are initialized for the simulations using the UMAT. In addition to a pure monotonic deformation, unloading of 0.1% and 0.5% at 1.5% axial strain, followed by reloading, was simulated. The axial strain $\varepsilon_{a}$ as a function of the axial stress $\sigma_{a}$ is shown in Fig. 4. Once again, the UMAT and the UDSM show similar results. The deviations from the original HSM are apparent, albeit small. More detailed analysis of the large unloading shows that the simulations with the UDSM deviate significantly from the original HSM in the low stress region and that the curve exhibits a kink. The same issue is also present in the UMAT, as the underlying implementation of the UMAT and the UDSM is the same and stems from Castellón [7].

### Boundary value problem

**Fig. 5:**
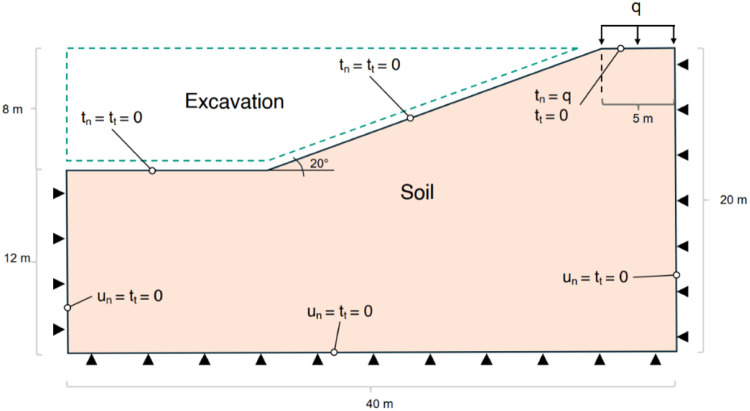
Geometry and boundary conditions of the BVP in the final calculation step.

To further assess the robustness of the proposed UMAT-UDSM interface, a boundary value problem (BVP) is considered, representing an excavated slope subjected to a surface loading. The model corresponds to a slope with a height of 8 m and an inclination of 20°, assuming plane strain conditions and quasi-static deformations. The geometry and the boundary conditions in the final calculation step are shown in Fig. 5. The soil represents dry Hostun RF sand with the parameters provided in Table 1.

**Fig. 6:**
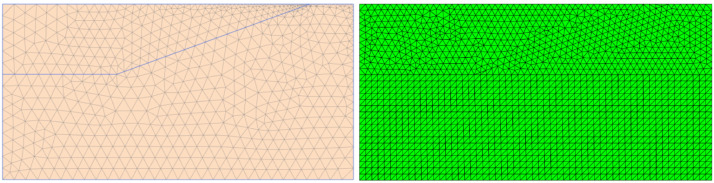
Finite element meshes used in Plaxis (left) and Abaqus (right).

**Fig. 7:**
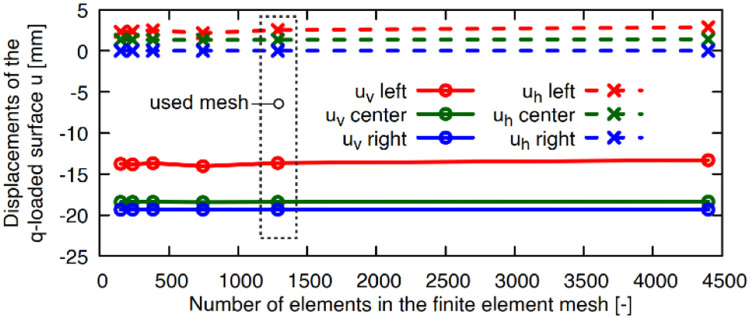
Final vertical and horizontal displacements at the left, center, and right point of the q-loaded surface for HSM simulations using finite element meshes with different element densities in Plaxis.

The analysis was conducted in three calculation steps: Step 1: Geostatic initialization under self-weight, assuming a unit weight of 19 kN/m^3^ and an isotropic stress state with $K_{0}\;=\;1$; Step 2: excavation of the slope by removing the upper 8 m of soil and Step 3: application of a uniform surface surcharge of q=80kPa kPa. The BVP is solved in Plaxis using the HSM and the UDSM implementation, as well as in Abaqus using the UMAT-UDSM interface (further called UMAT). The finite element meshes in Plaxis (1287 15-node triangular elements) and Abaqus (3348 CPE3 elements) are not identical, but have been generated carefully so that the solution is not affected by the mesh coarseness. The two meshes are shown in Fig. 6. Fig. 7 presents the final vertical and horizontal displacements of the q-loaded surface obtained from the HSM simulations in PLAXIS at three monitoring points (left, center, and right) for finite element meshes with varying numbers of elements. The results indicate that the model has been discretized with sufficient mesh density, as further mesh refinement produces only negligible changes in the computed displacements. This demonstrates that the solution has effectively reached mesh convergence and is therefore independent of the chosen mesh size.

The initialization of the additional state variables $\gamma_{p}$ and $p_{p}$ is not trivial, but it is of crucial importance, since the initial state has a decisive influence on the calculation results. By initializing the state variables accordingly, different overconsolidation ratios or, more generally, preloading histories can be considered in advanced soil models. To demonstrate the above-proposed UMAT-UDSM interface in BVP, the state variables must be initialized as identically as possible so that any differences in the calculation results are not primarily attributable to different initializations. While Plaxis performs this task internally for the HSM, the UDSM uses the equations defined by [7] using IDTask = 1. When using the UMAT in Abaqus, the additional state variables can be initialized spatially distributed using the subroutine sdvini.for. In the present UMAT simulations, the initial fields of $\gamma_{p}$ and $p_{p}$ were extracted from the UDSM simulations and initialized approximately using the subroutine sdvini.for. The initial fields of $\gamma_{p}$ and $p_{p}$ are therefore similar to each other in all calculations, although not identical.

**Fig. 8:**
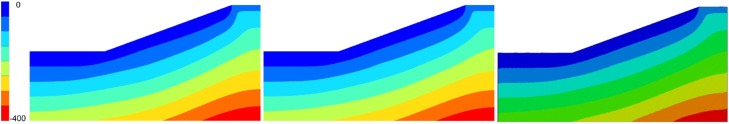
Resulting vertical stress component $\sigma_{v}$ [kPa] in the final step of the BVP using the HSM (left), UDSM (center), and UMAT (right) implementation.

**Fig. 9:**
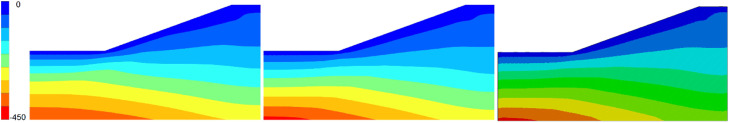
Resulting horizontal stress component $\sigma_{h}$ [kPa] in the final step of the BVP using the HSM (left), UDSM (center), and UMAT (right) implementation.

**Fig. 10:**
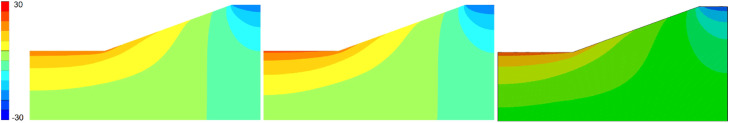
Resulting vertical displacements $u_{v}$ [mm] in the final step of the BVP using the HSM (left), UDSM (center), and UMAT (right) implementation.

**Fig. 11:**
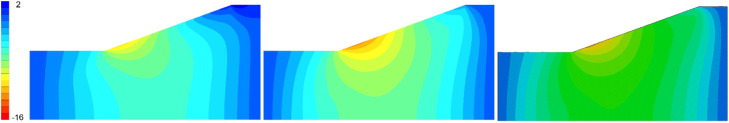
Resulting horizontal displacements $u_{h}$ [mm] in the final step of the BVP using the HSM (left), UDSM (center), and UMAT (right) implementation.

**Fig. 12:**
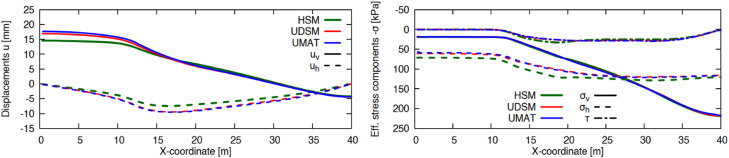
Displacements (left) and effective stress components (right) in a horizontal cross section one meter below the excavation level in the final step of the BVP using the HSM (green), UDSM (red), and UMAT (blue).

The calculation results are compared below as spatial distributions of displacements and stress components after the final calculation step. Fig. 8 shows the vertical stress component $\sigma_{v}$ and Fig. 9 illustrates the horizontal stress component $\sigma_{h}$. Fig. 10 displays the vertical displacement $u_{v}$ and Fig. 11 shows the horizontal displacement $u_{h}$. The horizontal and vertical displacements and the effective stress components in a horizontal cross-section situated one meter below the excavation level at the final stage of the BVP are presented in Fig. 12.

Once again, the calculation results are almost identical between the UMAT and the UDSM as well as the original HSM. The small deviations are due to uncertainties regarding initialization, as well as different meshes and different numerical solvers between Abaqus and Plaxis. It should also be noted that the simulations are performed with Hostun RF sand, for which the differences between the HSM and the UDSM were already most pronounced in the element simulations. However, in practical terms, the latter can be neglected.

The considered BVP thus confirms the feasibility of the proposed UMAT-UDSM interface and further provides a good approximation of the original HSM in Abaqus.

## Limitations

The present validation of the proposed UMAT-UDSM interface is limited to rate-independent constitutive models with internal state variables. Although the interface should, in principle, also work for rate-dependent formulations, a corresponding validation is still pending. The UMAT-UDSM interface has not yet been developed to address more complex issues such as partial saturation, thermal-mechanical coupling, etc., though this could be done in the future. The UMAT-UDSM interface requires a UDSM, which is available as a Fortran file. If only a dynamic link library (.dll file) is present, the interface cannot be used. Furthermore, numerical stability and computational efficiency may remain challenging, as the iterative coupling between the UMAT and the external UDSM may increase convergence sensitivity and runtime compared to native implementations.

## Credit author statement

**Luis Mugele:** Conceptualization Methodology, Software, Validation, Formal analysis, Investigation, Data Curation, Writing - Original Draft, Visualization, Supervision **Daniela Alejandra Villarreal Illada:** Conceptualization, Methodology, Software, Validation, Formal analysis, Investigation, Data Curation, Writing - Original Draft, Visualization **Heiko Neher:** Conceptualization, Resources, Writing - Review & Editing, Supervision **Christoph Niklasch:** Conceptualization, Resources, Writing - Review & Editing, Supervision **Hans Henning Stutz:** Conceptualization, Methodology, Resources, Writing - Review & Editing, Supervision, Project administration, Funding acquisition.

## Ethics statements

In this Manuscript, no human participants or animals, their data or biological material, are involved.

## Related research article

None.

## For a published article

None.

## Declaration of competing interest

The authors declare that they have no known competing financial interests or personal relationships that could have appeared to influence the work reported in this paper.

## Data Availability

Data will be made available on request.

## Footnotes

1 Note that for special analyses, for example under locally undrained conditions, the UMAT works with the total stress tensor provided by Abaqus.

2 Constitutive parameters are stored in the array props() and state variables are stored in the array statev() in Abaqus.

3 Constitutive parameters are stored in the array parms() and state variables are stored in the array stvar() in Plaxis.

4 To achieve consistency between Abaqus and Plaxis for time dependent constitutive models, the latter must be used with the same units. Plaxis operates using days, making the use of this unit preferable.
